# Emergency state in COVID-19 pandemic: Hungarian patients’ experiences

**DOI:** 10.1192/j.eurpsy.2021.838

**Published:** 2021-08-13

**Authors:** L. Pogany, A. Horváth, E. Rozsavolgyi, A. Slezák, J. Lazary

**Affiliations:** 1 Department Of General Psychiatry B, Nyírő Gyula National Institute of Psychiatry and Addictions, Budapest, Hungary; 2 Janos Szentagothai Doctoral School Of Neurosciences, Semmelweis University, Budapest, Hungary; 3 Ambulatory Mental Health Service, Zugló Health Service, Budapest, Hungary; 4 Department Of General Psychiatry D, Nyírő Gyula Institute of Psychiatry and Addictions, Budapest, Hungary; 5 Ambulatory Care Service, Nyírő Gyula Institute of Psychiatry and Addictions, Budapest, Hungary

**Keywords:** isolation, adaptation, pandemic, Fear

## Abstract

**Introduction:**

The COVID-19 pandemic made neccessary the declaration of emergency state in Hungary from 11 March 2020 to 18 June 2020. During this period hospitals were reserved for emergency use, ambulatory care was limited and replaced by telemedicine.

**Objectives:**

We assessed the opinions of patients of two ambulatory psychiatric care units in Budapest regarding the emergency state.

**Methods:**

We enrolled 438 outpatients in the survey (305 women and 133 men, mean age:51.9±16.2 years).Our questionnaire comprised 10 items on emotions and mental state and a 12 item „Problem evaluation scale”(included ’Fear’, ’Isolation’ and ’Health status’ subscales).General linear model (GLM), pairwise comparison and Tukey’s post hoc test were performed.

**Results:**

Up to 34% of patients considered that their condition worsened during this period, but 12% of them thought that this was not related to emergency state. Twice as many participants (12.8%) were concerned about their financial situation than about their health status (6.1%). Worsening health status, higher fear and more common adaptation difficulties were reported by patients < 50 years, than by subjects> 50 years (p=0.001; p=0.045; p=0.003). Isolation caused higher distress among women than in men (p=0.003). The abundance of information caused higher distress in patients with anxiety disorder than with psychotic disorders (p=0.024).Patients with affective disorders perceived higher vulnerability compared to subjects with psychotic disorders (p=0.004).

**Conclusions:**

Adaptation difficulties were reported by the half of the sample.Depletion of psychological resources can be expected during the next stage of the pandemic.

